# Laparoendoscopic single-site surgery in gynaecology: A new frontier in minimally invasive surgery

**DOI:** 10.4103/0972-9941.72387

**Published:** 2011

**Authors:** Amanda Nickles Fader, Kimberly L Levinson, Camille C Gunderson, Abigail D Winder, Pedro F Escobar

**Affiliations:** Divisions of Gynecologic Oncology, Greater Baltimore Medical Center and Johns Hopkins Medical Institutions, Baltimore, MD, USA; 1 Cleveland Clinic, Cleveland, OH, USA

**Keywords:** Conventional laparoscopy, laparoendoscopic single-site surgery, single-incision laparoscopic surgery, single port

## Abstract

**REVIEW OBJECTIVE:**

To review the recent developments and published literature on laparoendoscopic single-site (LESS) surgery in gynaecology.

**RECENT FINDINGS:**

Minimally invasive surgery has become a standard of care for the treatment of many benign and malignant gynaecological conditions. Recent advances in conventional laparoscopy and robotic-assisted surgery have favorably impacted the entire spectrum of gynaecological surgery. With the goal of improving morbidity and cosmesis, continued efforts towards refinement of laparoscopic techniques have lead to minimization of size and number of ports required for these procedures. LESS surgery is a recently proposed surgical term used to describe various techniques that aim at performing laparoscopic surgery through a single, small-skin incision concealed within the umbilicus. In the last 5 years, there has been a surge in the developments in surgical technology and techniques for LESS surgery, which have resulted in a significant increase in utilisation of LESS across many surgical subspecialties. Recently published outcomes data demonstrate feasibility, safety and reproducibility for LESS in gynaecology. The contemporary LESS literature, extent of gynaecological procedures utilising these techniques and limitations of current technology will be reviewed in this manuscript.

**CONCLUSIONS:**

LESS surgery represents the newest frontier in minimally invasive surgery. Comparative data and prospective trials are necessary in order to determine the clinical impact of LESS in treatment of gynaecological conditions.

## INTRODUCTION

Laparoscopy has become the standard treatment for many gynaecological conditions. Numerous studies have demonstrated that utilising laparoscopy for various benign and malignant gynaecological diseases results in shorter hospital stays, improved quality of life and improved surgical outcomes when compared with open abdominal surgery.[1–3] Laparoendoscopic single-site surgery (LESS), also known as single-port surgery, is a novel, rapidly-advancing minimally invasive technique. LESS improves the cosmetic benefits of minimally invasive surgery by providing only one incision, which also minimizes the potential morbidity associated with multiple incisions. Data from the general surgery and urology literature has demonstrated technical feasibility and reproducibility of this technique when utilised for a variety of procedures, including cholecystectomy, appendectomy, nephrectomy and hemicolectomy.[4–9] These early reports indicate that LESS is a promising surgical innovation that results not only in improved cosmesis, but also, in many cases, a shorter convalescence period and decreased postoperative analgesia requirements when compared with patients treated with conventional laparoscopic approaches.

Single-incision surgery has been performed anecdotally for decades in gynaecology; however, the first surgery employing LESS techniques was not described in the literature until 2008. Since that time, the interest in this surgical approach has grown exponentially. Several publications in the gynaecology literature have demonstrated preliminary feasibility, safety and reproducibility of LESS in the treatment of both benign and oncological female conditions.[10–20] The recent surge in the number and variety of LESS cases has been facilitated by the introduction of new instrumentation and access devices. In addition, innovative approaches to single-site surgery and utilisation of both new and existing robotic platforms have further improved and advanced single-site surgery.

## LESS TERMINOLOGY

Various terminologies and acronyms have been used to describe laparoscopic surgical procedures performed through a single-incision or surgical site [Table 1]. In 2008, an international consortium of minimally invasive experts (the Laparoendoscopic Single-Site Surgery Consortium for Assessment and Research--LESSCAR) met, with the goal of standardising the terminology for academic communications.[21] Review of the literature at that time by this group identified more than 10 frequently used terms to describe surgery through a single-incision. The conclusion of this consortium was to utilise the term ‘LESS’ surgery to describe all procedures performed in a minimally invasive manner through a single-incision. Furthermore, this consortium suggested that the term “U-LESS” be used to describe single-site surgery performed through the umbilicus. These terms have helped to improve research efforts by providing a universal language for these techniques. This not only allows search engines to be applied more efficiently to the developing literature, but also and allows clinical trials terminology to be standardised to promote a rapid dissemination of ideas and results.

**Table 1:** Categorization for laparoendoscopic single-site

Acronym	Full Procedure Name
LESS	Laparoendoscopic Single-Site Surgery*
OPUS	One-Port Umbilical Surgery
NOTES	Natural Orifice Transluminal Endoscopic Surgery
RSP	Robotic Single-Port Surgery
SILS	Single-Incision Laparoscopic Surgery
SIMIS	Single-Incision Minimally Invasive Surgery
SLIT	Single-Laparoscopic Incision Transabdominal Surgery
SPA	Single-Port Access
SPL	Single-Port Laparoscopy
SPICES	Single-Port incisionless conventional equipment utilising surgery
U-LESS	Umbilical Laparoendoscopic Single-Site Surgery

* Universal term selected by the international consortium Laparoendoscopic Single-Site Surgery Consortium for Assessment and Research--LESSCAR in 2008.[21].

## HISTORY OF LESS

Initially, during the 1960s and 1970s, the application of laparoscopic surgery in gynaecology was restricted to diagnostic cases and tubal procedures such as sterilisation.[18–20] The first reported case of laparoscopic organ resection was not until 1975, when Tarasconi, a Brazilian obstetrician/gynaecologist, preformed an endoscopic salpingectomy with these techniques.[22] As laparoscopic techniques evolved, gynaecologists began to perform the more complex cases of the specialty with these minimally invasive techniques. The use of LESS surgery in gynaecology evolved similarly, with initial utilisation for straightforward tubal ligations. For example, Wheeless *et al*.[23] performed thousands of tubal ligations using a single-puncture laparoscope with an offset eyepiece. Utilising this technique for more complex gynaecological surgery was first described by Pelosi, who performed a single-incision laparoscopic hysterectomy with bilateral salpingo-oophorectomy in 1991.[24] This method, however, was not popularized for another 15 years due to numerous procedural obstacles.

## BENEFITS OF LESS

Potential advantages of single-port over conventional multiport laparoscopy include superior cosmesis from a relatively hidden umbilical scar, a possible decrease in morbidity related to visceraland vascular injury during trocar placement, as well as risk reduction of postoperative wound infection, hernia formation and elimination of multiple trocar site closures. Studies have also suggested that women who undergo LESS report improved postoperative pain profiles when compared with those receiving conventional laparoscopic surgery.[25] This may be due to the utilisation of the umbilicus as the single site of the incision, as it is one of the thinnest regions on the abdominal wall, containing few blood vessels, muscle or nerves.

## LESS INSTRUMENTS AND TECHNOLOGY

LESS procedures are typically performed via one of two approaches. The first is single-site surgery, where two or more conventional ports are placed through a single-incision. The second approach utilises a single, multichannel device, through which multiple instruments and optics are passed. The access point for these surgeries is typically the umbilicus, although less cosmetic extra-umbilical incisions may occasionally be necessary to complete the surgery. Improvements in access devices, optics and instrumentation have driven the dissemination of this new format of laparoscopic surgery.

## SINGLE-PORT DEVICES AND INSTRUMENTATION

**Figure 1:**
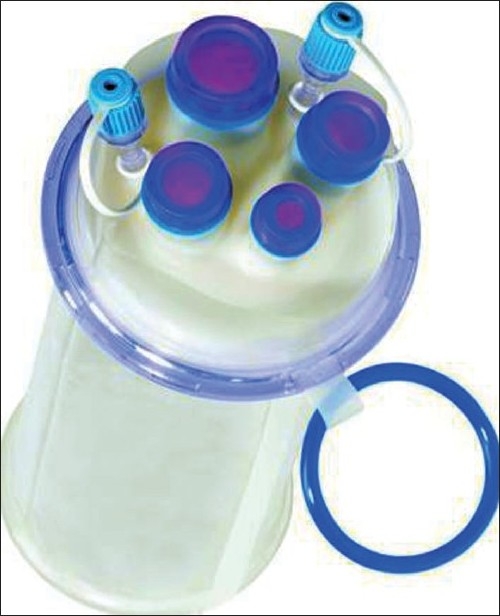
Quad Port (Advanced Surgical Concepts, Wicklow, Ireland).

LESS surgery can be performed through a variety of access devices. Conventional, low profile ports of different lengths can help to minimize limitation of movement by the surgeon(s) due to the extracorporeal interaction of the instruments and camera (known as “sword fighting”). Technological developments have produced multichannel single-port devices as well.[26–29] The prototype access system is the TriPort (Advanced Surgical Concepts, Wicklow, Ireland). This is a Food and Drug Administration-approved device with two components: a retracting component, which consists of an inner and outer ring with a double-barreled plastic sleeve; and a multichannel valve, which has three valves made of a unique elastomeric material. These valves each accommodate one 12-mm and two 5-mm instruments within the same working space. The port comes in various sizes, ranging from 10 to 30 mm, and can be selected according to the size of the fascial incision. More recently, the Quad-Port, which has four working channels [Figure 1] has been introduced. This port can accomodate one 15-, one 12-, and two 5-mm ports; or four 12-mm ports. While the TriPort can be placed by either an open approach or after insufflation with an introducer, the Quad-Port must be placed by an open-access technique.

Another port that has also been used successfully for LESS procedures is the Uni-X Single-Port Access Laparoscopic system (PNavel systems, Cleveland, OH). This port has three working channels for 5-mm instruments, and it is placed by an open access technique, requiring stitches to secure it to the fascia.

**Figure 2:**
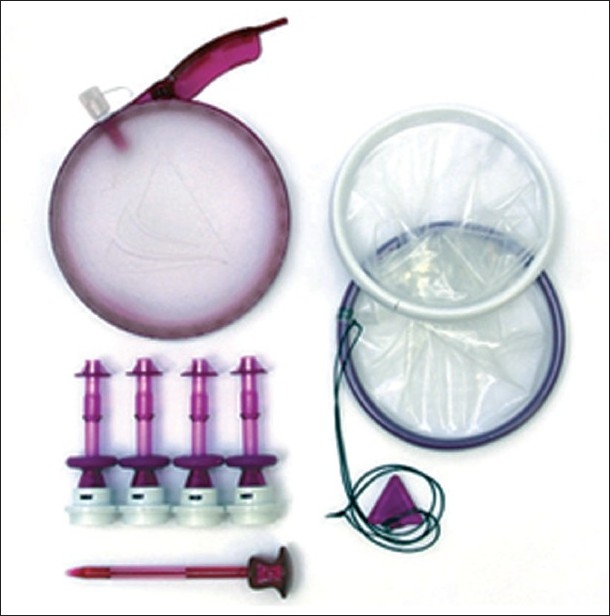
Gelpoint (Applied Medical Systems, Rancho Santa Margarita, CA).

The GelPort or newer generation GelPoint (Applied Medical, Rancho Santa Margarita, CA) devices consist of a combination of the rigid ring of the Alexis^®^ wound retractor with a Gelseal^®^ cap which maintains pneumoperitoneum during multiple instrument exchanges.[27,28] Unlike the aforementioned ports, this system allows for the introduction of ports or instruments of varying shapes and sizes directly through the gel [Figure 2]. This device can also be placed into a larger incision, allowing the surgeon to take advantage of the entire fascial incision required during extirpative procedures, and represents an efficient port for tissue removal. The disadvantage of this port can, however, is that it may balloon out during insufflation causing the instruments to be pushed further from the operative field and the fulcrum to be less stable than the other multichannel ports described.

**Figure 3:**
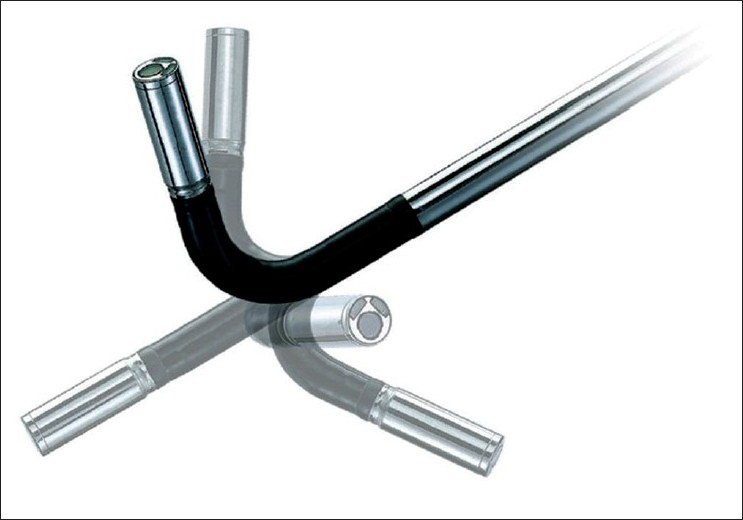
EndoEYE Deflecting Tip Laparoscope (Olympus America Inc, Center Valley, PA).

**Figure 4:**
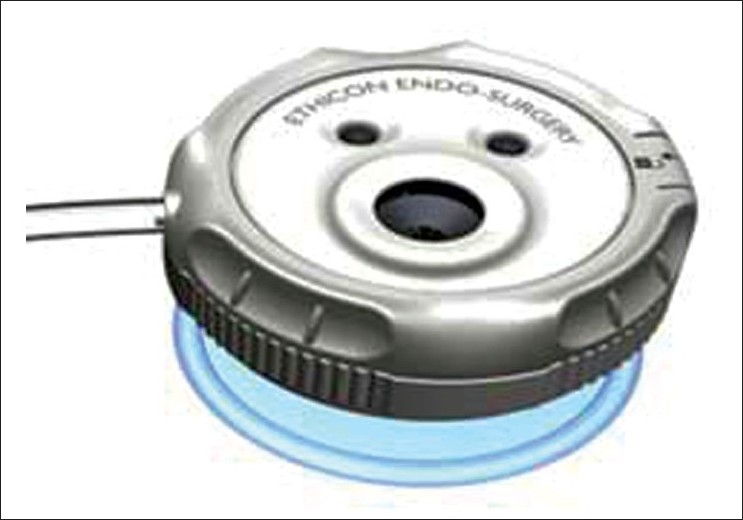
Single Site Laparoscopy Access System (Ethicon Endosurgery, Cincinatti, OH).

The SILS ^™^ Port Multiple Instrument Access Port (Covidien^®^, Mansfield, MA) is another multi-instrument access port that allows up to three laparoscopicinstruments (three 5-mm cannulas or two 5-mm and one 12-mm cannula) to be used simultaneously through separate flexiblechannels [Figure 3]. This port allows for adjustment of the cannula positions within the flexible port, and there is a separate channel that allows for CO _2_ insufflation. The port must be inserted through an open access technique. Finally, the latest port to be approved is the Single-Site Laparoscopy Access System (Ethicon Endosurgery, Cincinnati, OH) which was introduced on the market in 2010 [Figure 4]. Two 5-mm seals and one 15-mm seal enable use of a wide range of instrumentation. The advantages of this system include the use of a morcellator through the larger port; the integrated, low-profile system obviates the need for trocars; and the outer seal cap allows for 360° of rotation and reportedly facilitates specimen removal.

Two of the biggest caveats that limit use of the LESS technique are instrument crowding and lack of triangulation. Many surgeons are accustomed to the tenet of triangulation, due to the use of conventional laparoscopy, and this practice can be approximated to some degree by utilising special single-port instrumentation, including flexible and/or articulating instruments. These instruments allow for intracorporeal triangulation, rather than the extracorporeal triangulation utilised in traditional laparoscopy. Those instruments currently available as articulating devices includegraspers, shears, endoshears, needle-drivers, and hook electrocautery. Several companies have designed a comprehensive spectrum of these articulating instruments. At this stage in development, bulk and technical challenge pose major obstacles in using articulating instruments.

## OPTICS

**Figure 5:**
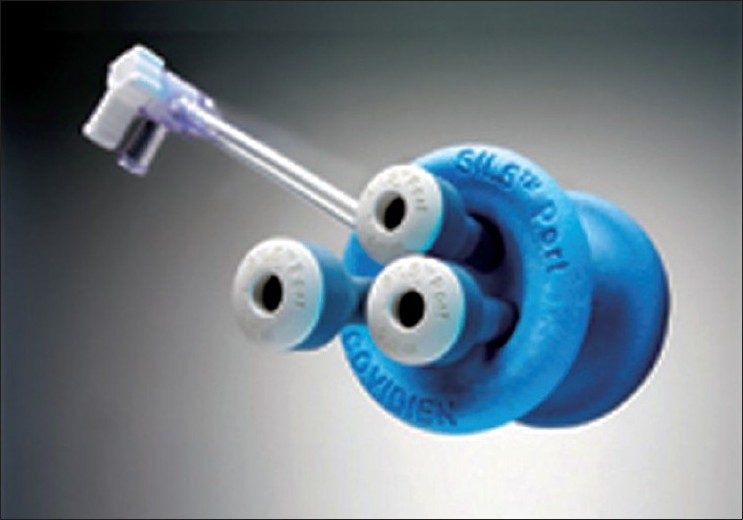
SILS Port (Covidien, Mansfi eld, MA).

Instrument crowding may occur due to several instruments being passed though a multichannel, single-port. Instruments may easily clash with one another or with the laparoscope, all of which are passed through the same surgical fulcrum. Conventional laparoscopes have a large extracorporeal profile with a light cable perpendicular to the telescope. Due to this latter feature, if the conventional laparoscope is utilised for LESS instrument clashing may be exacerbated. One method, however, to minimize this problem is by using a lower profile camera system. One system that is currently available is the rotatable 30°Visera EndoEYE laparoscope (Olympus America Inc, Center Valley, PA; Figure 5), which has the unique feature of a video laparoscope integrated with a coaxial light cable in line with the shaft of the telescope. The Olympus EndoEYE is currently the only laparoscope offering this unique feature, and it is available in 5-mm size in 0° and 30° configurations. It is also available with a flexible actively deflectable tip. Other special optics that may facilitate LESS surgery include a 45° telescope (Stryker), which has a coaxial, right-angle light guide adapter to help minimize sword fighting.

## PATIENT SELECTION

Although several patient and surgical factors can make minimally invasive surgery more challenging, variables such as obesity, previous abdominal surgeries and a diagnosis of early-stage gynaecological cancer), do not necessarily preclude the performance of LESS.[11,13,15] However, women who have had more than two previous midline vertical laparotomies, a prior panniculectomy or who do not possess a native umbilicus, or who have a diagnosis of advanced malignancy may not be suitable candidates for single-port laparoscopic surgery. Patient selection should initially be geared towards simple cases, as learning these ergonomically challenging techniques may be less technically demanding in these cases. Enrolling in a training/ simulation course is also helpful in order to become familiar with the newer LESS technologies. Both practicing techniques in a dry lab setting or working with and/or observing a more experienced LESS surgeon is also helpful when beginning to use LESS techniques. Further data is needed to clarify the optimal patient candidacy for this novel laparoscopic approach.

## REPORTS OF LESS IN BENIGN GYNAECOLOGY

Since the initial reports of clinical successes, there have been a large number LESS surgeries reported in gynaecology, with various new techniques being described. In 2005, Ghezzi *et al*.[18] described a “marionette-like” technique for treatment of tubal ectopic pregnancy using a single-incision; this was performed using a 10-mm operative laparoscope at the umbilicus and trans-abdominal suture retraction to steady the adnexa. Further advancements of this modality for treatment of ectopic pregnancy have been described in both case studies and cohort studies using single-site surgery with separate fascial punctures as well as single-port techniques.[19,20]

Both case reports and prospective studies have also described LESS in adnexal surgery for benign pathologies, including unilateral or bilateral salpingo-oopherectomy, adhesiolysis, excision of endometriosis and ovarian cystectomy.[12,30–33] These investigations suggest good results in terms of safety, cosmetics and postoperative pain. Some reports do, however, suggest that cystectomy is technically challenging due to difficulty in achieving the optimal traction-countertraction required for enucleation of cysts. Application of the LESS technique to hysterectomy has been described for both total hysterectomy (TLH) and laparoscopic-assisted vaginal hysterectomy (LAVH) via case reports and case-series.[13–17,34–37] LESS hysterectomy for uteri as large as 16-week size has been demonstrated with a variety of surgical approaches and methods of trocar placement, all of which have been shown to be safe and feasible. Several small case-control studies have compared single-incision approach LAVH to conventional multipuncture LAVH and have demonstrated similar operative characteristics including no significant difference in operative time, hospital stay or complications. Additionally, a recent retrospective cohort study by Yim *et al*. included 52 LESS hysterectomies compared with 101 conventional hysterectomies. This study demonstrated improved blood loss, hospital stay and pain scores (all *P* < 0.001) in women who underwent the LESS hysterectomy.[15] Further prospective trials are needed to confirm these results.

Recently, exceedingly small single-port devices have been explored in gynaecology. Chong *et al*. repoted on a series of 61 patients using a 2-mm miniport with standard trocar and instrumentation for the treatment of benign gynaecological disease. The results demonstrated that a 2-mm miniport is safe and feasible to use, and patient satisfaction was high, as greater than 80% of patients were satisfied with their surgical experience and 94% were satisfied with the cosmetic results.[38]

There is a learning curve for the techniques utilised in LESS single-port insertion and for total laparoscopic hysterectomy and bilateral salpingo-oophorectomy which was initially described by Fader and Escobar.[13] Studies examining the learning curve for performance of a Hasson open umbilical incision and single-port insertion show that the mean operative time to accomplish this task decreased significantly when comparing the first 1-10 cases (Quartile 1) vs. the next 11-20 cases (Quartile 2) (9.2 minutes versus 4.5 minutes). The time was even further decreased for cases 21-31 to 4.2 minutes (Quartile 3, *P* < 0.0001). Operative times for 31 consecutive LESS TLH/BSO cases showed similar improvements in operating time across all quartiles, with the most significant reductions observed between Quartile 1 cases (mean = 79.3 minutes) and Quartile 2 cases (mean = 55 minutes, *P* = 0.002). These preliminary results suggest a similar learning curve for conventional laparoscopy and LESS procedures to that of Hasson trocar placement.

## REPORTS OF LESS IN GYNAECOLOGICAL ONCOLOGY

In examining LESS techniques for gynaecological malignancies, several advantages may be proposed. Many women with breast or gynaecological cancers may perceive a benefit of LESS specific to their body image following surgery. Moreover, fewer incisions may result in faster recovery periods and a more timely administration of adjuvant therapies when required. More data is needed in order to support or refute these theories. There have been several reports from our research group demonstrating feasibility, safety and reproducibility of the LESS approach for treatment of select early-stage endometrial or ovarian cancers, pelvic masses, precancerous conditions and for risk-reducing salpingo-oophorectomy in women at high risk for ovarian cancer.[10,13] Furthermore, a recently report by Escobar *et al*. demonstrated that more complicated procedures, such as pelvic and para-aortic lymphadenectomy were also feasible and safe to perform with LESS techniques. These procedures produced comparable nodal counts to open or conventional laparoscopic surgery.[39] One limitation of LESS for nodal dissection was that visceral and truncal adiposity limited the access to the left para-aortic nodal region, and therefore, this procedure may be difficult to perform in morbidly obese woman utilising single-port techniques. One possible solution to this limitation is to position the patient in a lateral position with the left flank elevated (as is done with transperitoneal laparoscopic left nephrectomy procedures) in order to facilitate exposure of the left para-aortic lymph nodes to the level of the renal vessels. Further research is needed to determine if this positioning could provide adequate exposure in these patients.

In a retrospective review of 74 LESS cases performed on a gynaecological oncology service, a variety of procedures were performed (including endometrial cancer staging and excision of complex pelvic masses). This study showed that perioperative complications were low (3.5%) and pain scores were excellent.[13] A Pearson product-moment correlation demonstrated a significant linear relationship between the operating time and number of cases for cancer staging (r=-0.71, n=26, *P* < 0.001) and non-staging procedures (r=-0.78, n=48, *P* < 0.002). This statistic revealed that approximately 20 cases were required to achieve stable decreased operative times, again supporting a learning curve for complex LESS procedures that is similar to that of conventional laparoscopic cases.

Finally, LESS surgery may also be done via the da Vinci surgical system robotic platform through a GelPort or other similar single-port device.[11,40] Our group was the first to report on a robotic LESS gynaecological procedure utilising the S system. The newer technology of the generation SI da Vinci robotics (Intuitive Surgical) will have a specific single-port adapation that will allow for more facility with LESS. This may alleviate some of the challenging ergonomic issues associated with conventional LESS. The da Vinci single-port adaptation is currently undergoing FDA approval and will reportedly be available in 2011. This merger of single-port and robotics technology will further expand the use and feasibility of single-port surgery and is therefore eagerly anticipated.

## IS LESS THE FUTURE OF MINIMALLY INVASIVE SURGERY?

LESS surgery represents the next step forward in minimally invasive techniques; however, many questions must be answered and research must be performed to support the general application of LESS. There is definite patient interest, especially among women, as demonstrated by patients opting for single-port rather than multiport access surgeries; however, further research is need to answer the fundamental question of whether LESS shows any objective, reproducible benefit over conventional laparoscopy. There is agreement that these techniques provide improved cosmesis; however, standardized measures have not yet been employed to scientifically verify these findings. While this apparent benefit is important, the true indicator for the widespread acceptance of LESS will be whether or not these procedures demonstrates reduced pain, perioperative morbidity and convalescence thus justifying the increased technical demands and arguably more costly technology (when compared with conventional laparoscopy). Preliminary comparative studies indicate that LESS outcomes are at least equivalent to those of conventional laparoscopy, and in several studies LESS has shown improved pain profiles, decreased home oral narcotic requirements and faster return to work. It is likely that as the learning curve is overcome and large prospective studies are completed, that these questions will be addressed and more definitively answered.[41]

## CONCLUSION

Laparoendoscopic single-site surgery is the latest innovation in minimally invasive surgery and appears to be feasible and safe to perform for a variety of gynecologic conditions. This approach may result in a “scarless” effect, because the healed incision remains concealed at the base of the navel. While initial series in the urological, general surgery and gynaecological literature have demonstrated the safety, aesthetic superiority and potentially improved pain profile of a single-port approach when compared with conventional laparoscopy, these early findings must be further validated. The routine application of LESS in gynaecology not only requires evaluation of safety but also of cost-effectiveness, and these studies must all be performed in larger, prospective studies to definitively answer questions regarding the clinical and economic impact of this novel surgical approach. More detailed data is also needed to further describe the learning curve and the optimal environment for trainees and surgeons to become skilled in LESS procedures. These additional research initiatives will help to drive advances in minimally invasive surgical techniques and technology, in order to continue to provide better outcomes for our surgical patients.
